# Low Occurrence of *Salmonella* spp. in Wild Animals in Bahia, Brazil—Population Assessment and Characterization in the Caatinga and Atlantic Forest Biomes

**DOI:** 10.3390/ani14010021

**Published:** 2023-12-20

**Authors:** Eliege Jullia Eudoxia dos Santos, Amanda Teixeira Sampaio Lopes, Hllytchaikra Ferraz Fehlberg, Josiane Moreira Rocha, Pedro de Alcântara Brito Júnior, Fernanda Coelho Simas Bernardes, Thaise da Silva Oliveira Costa, Elisa Arcanjo Guilherme, Kristel Myriam De Vleeschouwer, Leonardo de Carvalho Oliveira, Beatris Felipe Rosa, Beatricy Silva de Amorim, Leildo Machado Carilo Filho, Elson Oliveira Rios, Suelen Sanches Ferreira, Dália dos Prazeres Rodrigues, George Rêgo Albuquerque, Flávia Regina Miranda, Martin Roberto Del Valle Alvarez, Victor Goyannes Dill Orrico, Rachel Passos Rezende, Selene Siqueira da Cunha Nogueira, Lucas Jose Luduverio Pizauro, Bianca Mendes Maciel

**Affiliations:** 1 Graduate Program in Animal Science, Santa Cruz State University, Ilhéus 45662-900, BA, Brazil; eliegejullia@hotmail.com (E.J.E.d.S.);; 2 Undergraduate Program in Veterinary Medicine, Santa Cruz State University, Ilhéus 45662-900, BA, Brazil; 3 Centre for Research and Conservation, Royal Zoological Society of Antwerp, B-2018 Antwerpen, Belgium; 4 Bicho do Mato Research Institute, Belo Horizonte 30360-082, MG, Brazil; 5 Graduate Program in Zoology, Santa Cruz State University, Ilhéus 45662-900, BA, Brazil; 6 Mammals Collection Alexandre Rodrigues Ferreira (CMARF-UESC), Santa Cruz State University, Ilhéus 45662-900, BA, Brazil; 7 North Fluminense Foundation for Regional Development, Campos dos Goytacazes 28053-100, RJ, Brazil; 8 Enterobacteria Laboratory, Oswaldo Cruz Institute, Rio de Janeiro 21041-250, RJ, Brazil; 9 Department of Agricultural and Environmental Sciences, Santa Cruz State University, Ilhéus 45662-900, BA, Brazil; 10 Department of Biological Sciences, Santa Cruz State University, Ilhéus 45662-900, BA, Brazil

**Keywords:** biodiversity, epidemiology, environment, multidrug resistance, public health, wildlife

## Abstract

**Simple Summary:**

This study evaluated the possible role of wildlife in the Atlantic Forest and Caatinga biomes of Bahia, Brazil, as reservoirs of *Salmonella*. Very low frequencies (4/674 = 0.59%) of *Salmonella* infections and antibiotic resistance were observed. Thus, the findings of this study indicated that a wide variety of wildlife species do not carry *Salmonella*. This may be attributed to minimal human interference. Bacteria of potential public health concern were only detected in areas with high human interaction; therefore, we propose that *Salmonella* may be a good indicator of degradation in wildlife environments.

**Abstract:**

*Salmonella* spp. are known to persist in the environment. Wild animals are believed to act as important reservoirs, with antimicrobial resistance frequently occurring in the environment. However, little is known about the role of the wildlife in Bahia as a reservoir for *Salmonella* in Brazil. This study aimed to isolate and characterize *Salmonella* spp. from wildlife in the Atlantic Forest and Caatinga biomes considering indicators such as the animal species, degree of anthropization, sampling area, and feeding habits. Convenience wildlife sampling and characterization were conducted, followed by microbiological and molecular identification of *Salmonella* isolates, serotyping, and antimicrobial susceptibility testing. A total of 674 fecal samples were collected from 12 municipalities during 2015–2021, and 4 were positive for the following *Salmonella* species: *Salmonella enterica* subspecies *enterica* serovar Agona (*n* = 1), *Salmonella enterica* subsp. *enterica* serogroup O:16 (*n* = 2), and *Salmonella enterica* subsp. *enterica* serovar Muenchen (*n* = 1). Antimicrobial susceptibility analysis revealed that one isolate was resistant to six antibiotics, including extended-spectrum penicillins and beta-lactamase inhibitors. These results indicated a low frequency of *Salmonella* spp. in the sampled forest fragments. The presence of *Salmonella* in wild animals increases the risk to public health and biodiversity and indicates that they can act as sentinels of environmental contamination or indicators of preservation.

## 1. Introduction

The current concept of health is extended to the idea of “One World, One Health” as it considers the tripartite relationship between humans, animals, and the environment [1]. In this regard, wildlife is considered to play an important role as sentinels for the detection of infectious diseases related to the impact of anthropogenic actions and by taking an active role in transmitting pathogens to other animals and humans [2]. Anthropogenic influences in forest areas occur due to demographic expansion related to economic activities, which generates waste that contaminates ecosystems and consequently alters the ecological relationships between species in different habitats [3]. This combination of factors directly and indirectly promotes environmental and health changes in biomes and the agent/host/disease relationship [4].

Contact with wild fauna can increase the occurrence and/or incidence of pathogens both unidirectionally and bidirectionally [5], allowing organisms limited to certain areas and species to be transported to others [6]. This relationship intensifies if commercial animal breeding sites are located close to forested areas, as this promotes the sharing of food, water, and shelter [7]. Wild animals play an important role in the epidemiology of *Salmonella* spp. because they act as asymptomatic latent carriers of various serovars that can be isolated from a wide variety of wild animals [8] and survive in aquatic environments for long periods [2]. Infectious pathogens of wildlife origin have recently gained attention and are considered important globally because of their role in livestock health and productivity [7].

*Salmonella* spp. can persist in the environment, facilitating infection of wild animals. Antimicrobial resistance frequently occurs in the environment, directly affecting populations of domestic and wild animals [9]. The literature indicates that *Salmonella enterica* resistance has intensified because resistant organisms encode virulence and resistance genes [10] that are transported inter- and intraspecies via the horizontal transfer of integrons and/or plasmids [11]. In addition, the zoonotic nature of *Salmonella* spp. has been observed in outbreaks in which strains from wild animals showed little genetic variance compared with serovars isolated from humans [12]. Moreover, isolates from wild animals show phylogenetic similarities with those from domestic animals and food [13].

Thus, this study aimed to isolate and characterize *Salmonella* spp. from wildlife sampled from the Atlantic Forest and Caatinga biomes in Bahia, Brazil, considering indicators such as the animal species, degree of anthropization, sampling area, and feeding habits. This is essential because there is currently limited knowledge about the role of wildlife as a reservoir of this agent in the sampled regions.

## 2. Materials and Methods

### 2.1. Ethical Approval

The following components of this study were approved by the Ethics Committee on Animal Use of the State University of Santa Cruz (CEUA-UESC) and the Oswaldo Cruz Institute (IOC): the use of traps to capture wild birds in 2016–2017 (CEUA-UESC protocol code: 014/2014; date of approval: September 2014); the use of traps to capture wild mammals in 2015 (CEUA-UESC protocol code: 003/2013; date of approval: May 2013), 2017 (CEUA-UESC protocol code: 018/2015; date of approval: August 2015), 2018–2020 (CEUA-UESC protocol code: 028/2018; date of approval: November 2018), and 2021 (CEUA-IOC 036/2020; date of approval: March 2021); and the use of traps to capture wild reptiles in 2018 (CEUA-UESC protocol code: 034/16; date of approval: September 2016).

### 2.2. Study Area

**Figure 1:**
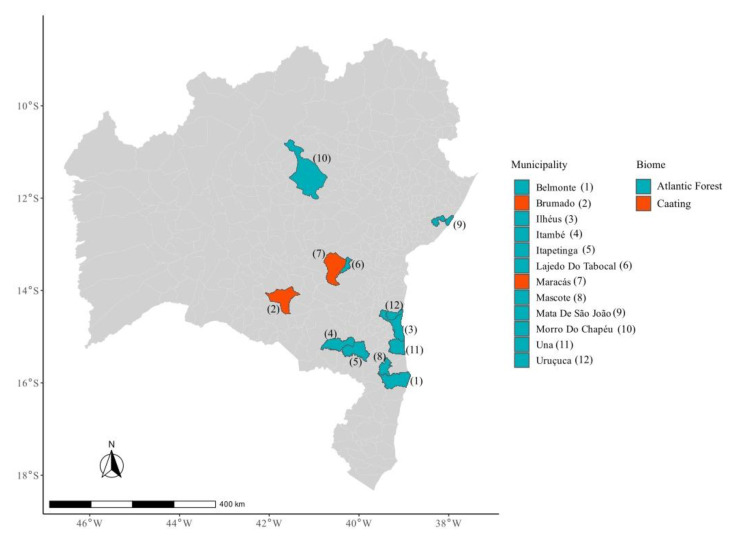
Biome of areas where the wildlife was captured and sampled for *Salmonella* screening from 2015 to 2021 in Bahia State, Brazil.

Bahia is located in northeastern Brazil, covering a total area of 564,760,429 km^2^, with a population of approximately 14,136,417. This study was conducted in two biomes corresponding to 12 municipalities in the state of Bahia, Brazil: the Atlantic Forest (Belmonte, Ilhéus, Itambé, Itapetinga, Lajedo do Tabocal, Maracás, Mascote, Mata de São João, Morro do Chapéu, Una, and Uruçuca) and Caatinga (Brumado and Maracás) biomes (Figure 1). The areas were classified according to their biomes and vegetation types according to the Brazilian Institute of Geography and Statistics [14]. A comprehensive assessment was conducted based on predefined indicators to evaluate the degree of anthropization in each study area. These included land-use intensity, vegetation cover, biodiversity, and the prevalence of human activities. These categories of land cover and land use were obtained using MapBiomas software v7.1 “https://mapbiomas.org/en (accessed on 28 August 2023)”, from raster images of 30 m definition. Their percentages in each buffer were calculated in R software v4.1.1 using the “extract” function of the raster package. A buffer radius of 1 km was used around the collection sample points. The anthropization was considered as the sum of the percentages of the areas with pasture, other cultures, silviculture, and urbanization.

### 2.3. Specimen Collection

The orders Didelphimorphia and Rodentia were captured with falling traps (bucket size of 60 L and arranged 10 m from each other), Sherman traps (31 × 8 × 9 cm), and Tomahawk traps (46 × 16 × 17 cm) arranged in transects and with bait comprising a mixture of sweet peanut, cornmeal, sardine, and banana. In 2015, the municipalities of Belmonte (June and July, *n* = 47), Mascote (August, *n* = 19), and Una (August, *n* = 15) were sampled for 10 consecutive nights, with a total of 13,020 traps/night. Sampling was conducted in the Itapetinga and Itambé municipalities for seven days each in August 2018 and February 2019, using 7742 traps/night. Similarly, in Lajedo do Tabocal and Maracás, sampling occurred for 10 days each in August 2019 and February 2020, using 6820 traps per night.

After collection for a previous research project, small mammals were euthanized by intramuscular injection of 100 mg/kg ketamine hydrochloride + 5 mg/kg xylazine hydrochloride (rodents) or 30 mg/kg ketamine hydrochloride + 2 mg/kg xylazine hydrochloride (marsupials) in the gluteal region and deposited as witness material in the Alexandre Rodrigues Ferreira collection of mammals belonging to the State University of Santa Cruz (CMARF/UESC). The collected individuals were used for taxonomic and morphological characterization and demonstration of the sexual dimorphism of the species. However, pups and pregnant and lactating females were identified using numbered aluminum earrings and returned to the place of capture. 

Samples were collected from golden-headed lion tamarins, *Leontopithecus chrysomelas*, in 2017 (March, May, June, and July) from Ilhéus (*n* = 12) and Una (*n* = 9). The collection method involved the use of Tomahawk traps measuring 48.3 cm long × 15.2 cm wide × 15.2 cm high, baited with bananas. These traps were deployed for approximately 11 h daily, accounting for 22 h per month at each location. After capture, *L. chrysomelas* were anesthetized with intramuscular ketamine 10 mg/kg and midazolam 0.3 mg/kg.

The Atlantic Forest endemic sloth, *Bradypus torquatus*, was sampled in 2021 (May and October) in Mata de São João (*n* = 11) using an active search. When the maned sloth was located, a trained climber accessed the treetops to catch the animals using their hands. During the physical restraint, the claws of *B. torquatus* were wrapped with Velcro tape to keep them closed. The animal was placed in a cotton bag and lowered to the forest floor using a rope. The animals were sedated with 4.0 mg/kg ketamine hydrochloride and 0.03 mg/kg medetomidine hydrochloride intramuscularly and 0.1 mg/kg atipamezole hydrochloride intravenously.

In the municipalities of Uruçuca, Ilhéus, and Una, wild birds were captured using mist nets, with each net active for approximately 10 h daily and inspected every 40 min. Ilhéus was sampled in 2016 (November) and 2017 (April and June) and Una in 2016 (November) and 2017 (March and August), with a sampling effort of 40,500 h·m^2^ for five consecutive days. Sampling in May and June 2017 in Uruçuca resulted in a sampling effort of 32,400 h·m^2^ for four consecutive days.

Manual collection of the Reptilia class was performed using an active search methodology in 2018 (September and October) in Brumado (*n* = 11), Ilhéus (*n* = 1), and Morro do Chapéu (*n* = 4) between 6 p.m. and 11 p.m. without standardization.

Fecal samples were collected using rectal swabs from 674 wild animals (Table S1) captured from forest remnants and adjacent pastures in the Atlantic Forest (*n* = 548) and Caatinga (*n* = 126). Wildlife genera and species were defined based on morphological and anatomical criteria. Upon collection, all swab samples were kept in 2 mL microtubes containing 1 mL of buffered peptone water (Liofilchem, Roseto degli Abruzzi, Italy) and promptly refrigerated to maintain their integrity during transportation to the laboratory for further processing.

### 2.4. Salmonella Isolation and Identification

*Salmonella* samples from avians used in this study [15] and from mammalian and reptilian samples were obtained as follows. Rectal swab samples underwent a pre-enrichment step, which was performed in 2 mL microtubes containing 1 mL of buffered peptone water (Liofilchem, Roseto degli Abruzzi, Italy) at 37 °C for 24 h. Thereafter, selective enrichment was performed by inoculating 1 mL of the bacterial suspension in 9 mL of Rappaport Vasiliadis broth (Oxoid Ltd., Basingstoke, UK) and incubating at 43 °C for 18–24 h. Differential isolation was then performed by streaking a loopful on xylose-lysine-deoxycholate agar (Acumedia; Neogen Corporation, Lansing, MI, USA) and Hektoen enteric agar (Becton Dickinson, Franklin Lakes, NJ, USA) and incubating at 37 ± 1 °C for 18–24 h. Suspected colonies were inoculated in 1 mL of trypticase soy broth (HiMedia Laboratories Pvt. Ltd., Mumbai, India). The samples were then incubated (37 °C/18–24 h) for presumptive biochemical identification using triple sugar iron agar (HiMedia Laboratories Pvt. Ltd.), lysine iron agar (HiMedia Laboratories Pvt. Ltd.), urease (Acumedia, Neogen Corporation), and strips of oxidase (Laborclin, Pinhais, Brazil).

Presumptive colonies of *Salmonella* spp. were grown in trypticase soy broth (HiMedia Laboratories Pvt. Ltd.) at 37 °C for 18–24 h before submitting to DNA extraction using a DNA PureLink™ Genomic DNA Mini Kit (Invitrogen, Carlsbad, CA, USA), according to manufacturer instructions, and were confirmed using polymerase chain reaction (PCR), as described below. The total PCR reaction was 25 µL and consisted of 1× PCR buffer (Invitrogen, Carlsbad, CA, USA); 1.25 mM MgCl_2_ (Invitrogen); 200 µM dNTP; 10 pmol of each primer, ST11 (5′-AGCCAAACCATTGCTAAATTGGCGCA-3′) and ST15 (5′ TTTGCGACTATCAGGTTACCGTGG-3′), which are specific for the *Salmonella* genus [16]; 1.25 U of Taq DNA polymerase (Invitrogen); and 2 µL of DNA (50 ng/µL) from the suspected colony of the pathogen. Sterile nuclease-free water was used to complete the reaction volume to 25 µL. *S. enterica* serovar Enteritidis PT1 was used as a positive control, and a DNA-free reaction was used as a negative control. The amplification occurred with an initial denaturation (5 min at 94 °C) followed by an amplification phase of 35 cycles (30 s at 94 °C, 30 s at 62 °C, and 1 min at 72 °C) and a final extension step (7 min at 72 °C) in a Proflex PCR system (Applied Biosystems; Life Technologies, Carlsbad, CA USA). PCR products were visualized on a 1% agarose gel stained with SYBR Green (Invitrogen) and examined under UV light [17]. The PCR-positive samples were further serotyped using serogroup- and serovar-specific antisera obtained from the Enterobacteriaceae Laboratory of the Oswaldo Cruz Foundation in Rio de Janeiro, Brazil.

### 2.5. Antibiotic Susceptibility Test

Antimicrobial resistance was assessed using the Kirby–Bauer disk-diffusion method on Mueller–Hinton agar (HiMedia Laboratories Pvt. Ltd.), with the bacterial suspension turbidity adjusted to a McFarland scale of 0.5 [18], and following the Clinical Laboratory Standards Institute [19] protocol. The following 15 antimicrobial compounds were used: amikacin (30 µg), amoxicillin/clavulanic acid (20/10 µg), ampicillin with sulbactam (10/10 µg), cefepime (30 µg), cefoxitin (30 µg), ciprofloxacin (5 µg), chloramphenicol (30 µg), gentamicin (10 µg), imipenem (10 µg), lomefloxacin (10 µg), norfloxacin (10 µg), piperacillin with tazobactam (100/10 µg), sulfazotrim (trimethoprim sulfamethoxazole (25 µg)), tobramycin (10 µg), and trimethoprim (5 µg) (LABORCLIN, Produtos para Laboratórios Ltd., Pinhais, Brazil). *Escherichia coli* ATCC 25922 was used for quality control.

### 2.6. Statistical Analysis

Due to the limited number of positive samples, only descriptive statistical analyses were performed [20]. In addition, the patterns of wild animals (diet, locomotion, species, biomes, and municipalities) were analyzed using descriptive and inferential statistics [20].

## 3. Results

Between 2015 and 2021, 674 samples were collected from wild animals in the Atlantic Forest and Caatinga. These samples were obtained from the 12 municipalities studied and were collected from the following animals: 114 from birds, 544 from mammals (317 rodents, 195 marsupials, 21 primates, and 11 sloths), and 16 from reptiles (Table S1). Different study areas showed varying land use and, consequently, different percetanges of anthropization. The forest environments studied showed lower percentages of anthropization (except for Itapetinga and Mascote) when compared with Caatinga (except for Brumado). It was not possible to observe if areas that had anthropization such as the cocoa plantations and agroforestry systems had any influence on biodiversity. Four Salmonella isolates (0.59%) were obtained from three mammals and one bird. These isolates belonged to three distinct serovars: *S. enterica* subsp. *Enterica* serovar Agona, *Salmonella enterica* subsp. *enterica* O:16, and *Salmonella enterica* subsp. *enterica* serovar Muenchen. All positive samples were found in areas of the Atlantic Forest (Table 1) and had percentages of anthropization of 51.8%, 47.0%, 79.2%, and 70.3% (Table S2). All negative *Salmonella* samples varied in their anthropization percentages (Table 2).

The antimicrobial sensitivity profile of *Salmonella enterica* subsp. *enterica* serogroup O:16 isolated from a young female *Didelphis albiventris* showed resistance to 6 of the 15 antimicrobials tested (amoxicillin/clavulanic acid, ampicillin with sulbactam, cefoxitin, chloramphenicol, trimethoprim, and sulfamethoxazole). The other serovar isolated from an adult female did not exhibit antimicrobial resistance. *S.* Agona isolated from a female passerine showed intermediate resistance to amikacin and gentamicin. *S*. Muenchen from a male *Cerradomys vivoi* demonstrated intermediate resistance to amikacin. 

The most frequent family was Cricetidae (282), followed by Didelphidae (197), Pipridae (34), Turdidae (32), Echimidae (30), Dendrocolaptidae (24), Callithrichidae (21), Bradypodidae (11), Sphaerodactylidae (11), Columbidae (6), Thraupidae (5), and Tyraniidae (4). The other families were represented by fewer than 2 exemplars and were thus grouped as others (17).

The most frequently observed species were Didelphis albiventris (95), Necromys lasiurus (68), Calomys expulsus (62), Cerradomys vivoi (44), Marmosops incanus (39), Hylaeamys seuanezi (32), Gracilinanus agilis (31), Rhipidomys mastacalis (23), Wiedomys pyrrhorhinos (23), Leontopithecus chrysomelas (21), Turdus leucomelas (18), Thrichomys aff laurentius (18), Marmosa murina (16), Turdus rufiventris (13), Ceratopipra rubrocapilla (12), Bradypus torquatus (11), Coleodactylus meridionalis (11), Dixiphia pipra (9), Xiphorhynchus fuscus (8), Oligoryzomys nigripes (8), Trinomys albispinus (8), Dendrocincla turdina (7), Manacus manacus (7), Mus musculus (7), Oligoryzomys stramineus (7), Machaeropterus regulus (6) Cryptonanus agricolai (6), Leptotila rufaxilla (5), Glyphorynchus spirurus (4), Trinomys sp (4), and Monodelphis americana (3). Other species, which were represented by fewer than 2 exemplars each or were not identified, were grouped as others (48) (Table 3).

Most of the wild animals studied were from the Atlantic Forest biome (79.5%), were terrestrial (40.5%), and had a mostly fruit-based diet (71.6%), and 56.8% were collected during the rainy season (Table S1). When comparing animal class, season, biome, animal locomotion, and animal diet, most were terrestrial mammals fed a fruit diet (32.9%) (Table S1). A detailed assessment of the diet, locomotion, and biome is provided in the Supplementary Material (Table S2). The municipalities with the highest numbers of animals were Maracás (175), followed by Itapetinga (122), Lajedo do Tabocal (80), Una (72), Ilhéus (62), Itambé (54), Belmonte (47), Mascote (19), Uruçuca (17), Brumado (11), Mata de São João (11), and Morro do Chapéu (4).

The most frequently observed feeding behaviors were frugivore/omnivore (187), frugivore/granivore (174), insectivore/omnivore (105), frugivore/insectivore (46), frugivore (34), insectivore (33), frugivore/seed predator (24), granivore (18), frugivore/invertivore (13), folivore (11), and invertivore (11) (Table S3).

## 4. Discussion

The results observed in this study may not be directly comparable with those of other studies because the actual prevalence of *Salmonella* spp. depends on the sampling location and methods applied [21]. Thus, the low prevalence (0.59% in the 6-year convenience sampling) of *Salmonella* spp. may be attributed to cloaca/rectal swab sampling. However, in a previous Danish study on carcasses, the authors observed a low prevalence of *Salmonella* spp. in wild birds [22] and boars [9]. Similarly, a low frequency of isolation has been reported in wild birds from a Swiss rehabilitation center (1.08%) [22] and in wild animals in northwestern Italy (4.30%), including canids, mustelids, wild birds, and ungulates [23]. In addition, another study from western Italy reported a frequency of 2.87% in wild animals, such as magpies, foxes, crows, jays, brown hares, hedgehogs, starlings, swans, wild ducks, Porcupine’s green woodpeckers, and Eurasian collared doves [7]. No isolates of *Salmonella* spp. were obtained in a study on wild rodents (*n* = 237) in Poland [24]. These results reinforce the fact that the prevalence of *Salmonella* spp. is usually lower in wild than in domesticated animals [25].

A low frequency of positive results (9.5%) was observed in the wild boar population in São Paulo [9]. These results were attributed to the fact that as the number of human settlements in close vicinity increases, wild animals come into contact with other animal species, raw food, and high temperatures, all of which favor the occurrence of *Salmonella* spp.; therefore, they can serve as vectors for pathogen transmission [26]. This behavior was observed in a previous study [27], where rodents from urban areas were more prone to carry pathogens. Therefore, as stated by Skov [21], the prevalence of *Salmonella* spp. in wildlife can vary greatly among studies, and this variation is not associated with sampling methods, study designs, or differences in prevalence between countries.

Although the areas sampled were exposed to human contact, the low occurrence of *Salmonella* spp. in this study may be attributed to the fact that the areas were highly conserved and animals from preserved areas showed a lower risk of contamination than those with human contact [26]. In addition, the animals presented a mostly terrestrial and frugivorous nature, which may contribute to their occurrence because the behavior and feeding habits of wild animals are known to influence the likelihood of infection with *Salmonella* [27]. Badgers, red foxes, raptors, and wild boars acquire *Salmonella* infections by scavenging contaminated carcasses or human leftovers [8]. However, wild animals can be latent carriers and thus contribute to the low frequency of isolation because, during some periods, the microorganisms are located intracellularly and are not released into the environment [28].

All positive samples were obtained from the Atlantic Forest. This result agrees with that of another study on 36 wild mammals from the Atlantic Forest and Amazonia biomes in Brazil, which obtained only one isolate of *Salmonella enterica* subspecies *diarizonae* serotype 60:r:e,n,z15, which was isolated from a rodent in Amazonia [2]. In another study of 207 wild rodents from farmland and nonfarmland areas in Sweden, only one *Salmonella enterica* serovar Typhimurium isolate from a subpopulation of 11 individuals was obtained from a chicken farm [29]. In our study, the samples positive for *Salmonella* spp. from small mammals were from areas close to a cattle farm, which is a factor alongside the changes made to the forest area as it favors the habit of looking for shelter and food in peridomiciliary areas, thus facilitating the acquisition and dispersion of pathogenic microorganisms [30]. Among the marsupials sampled in this study, two *Didelphis albiventris* tested positive for *Salmonella enterica* subsp. *enterica* O:16. Similar results were found when *Didelphis albiventris* in a rural area in Argentina (five poultry farms) and eight surrounding forest areas were sampled, and a 4% positivity rate for *Salmonella enterica* was found. However, the authors found no relationship between age and sex [31]. Additionally, Casagrande [32] isolated *Salmonella* from 17.0% (18/106) of *Didelphis aurita* and 17.5% (7/40) of *Didelphis albiventris* individuals in their study, most of which were wild animals that lived in the forest around a human population. These authors also found no influence of sex or age on the frequency of *Salmonella* infections.

Resistance to environmental bacteria is a natural biological process, and its capacity for transmission and dissemination is enhanced by multiple drug resistance genes shed in the feces of livestock and humans [33]. However, fragmentation promotes environmental stress in ecosystems, allowing the selective pressure of multiresistant agents on antibiotics [34]. With interactions between humans, domestic animals, wild animals, and the environment, contact has intensified, increasing the chances of transmission of resistant strains [35]. Therefore, free-ranging wild animals that have not been exposed to antibiotics may have high rates of drug resistance owing to environmental contamination [36]. This resistance can also be acquired from consuming contaminated water or food and transported via direct contact with garbage and sewage residues [37].

The detection and analysis of microorganisms, mainly bacteria, can provide information on ecosystem exposure to biological agents [38]. Based on our findings, we propose that the detection of pathogenic *Salmonella* spp. isolates be used for biomonitoring environmental quality in future wildlife studies. This approach facilitates the assessment of conservation and management practices.

## 5. Conclusions

In the present study, wild animals assessed in the mesoregions showed a low frequency (4/674 = 0.59%) of *Salmonella*, which is consistent with previous reports on wild animal populations worldwide. In addition, the isolates were found to be multiresistant, including resistance to extended-spectrum penicillins with beta-lactamase inhibitors. Thus, it is important to consider that specific behavior, diet, and the low percentage of anthropization may be the main factors affecting the studied species in the Atlantic Forest and Caatinga in Bahia, Brazil. Therefore, we propose that the detection of *Salmonella* spp. can be used for anthropization biomonitoring and as an indicator of environmental preservation in future wildlife studies.

## Figures and Tables

**Table 1:** Characterization of the captured and sampled wildlife positive for *Salmonella* spp. according to their respective location and environment.

Biome	Age	Sex	Municipality	Season	Sampling Date	Anthropization (%)	Serovars	Species	Class
Atlantic Forest	Adult	F	Ilhéus	Rainy	27 July 2017	51.8	Agona	*Ceratopipra* *rubrocapilla*	Avian
Atlantic Forest	Adult	F	Itapetinga	Dry	24 August 2018	47.0	O:16	*Didelphis* *albiventris*	Mammalia
Atlantic Forest	Young	F	Itapetinga	Rainy	5 February 2019	79.2	O:16	*Didelphis* *albiventris*	Mammalia
Atlantic Forest	Adult	M	Itambé	Rainy	7 February 2019	70.3	Muenchen	*Cerradomys* *vivoi*	Mammalia

**Table 2:** Characterization of the captured and sampled wildlife negative for *Salmonella* spp. according to their respective location, biome, and anthropization percentages by wildlife family and by municipality.

Municipality	Family (*n*)	Biome	Anthropization (%) by Family	Anthropization (%) by Municipality
Belmonte	Cricetidae (33)	Atlantic Forest	39.8 ± 1.51	39.9 ± 1.54
	Didelphidae (12)		40.6 ± 1.62	
	Sciuridae (2)		38.8 ± 0.00	
Brumado	Sphaerodactylidae (11)	Caatinga	17.2 ± 0.00	17.2 ± 0.00
Ilhéus	Callithrichidae (12)	Atlantic Forest	21.7 ± 0.00	46.2 ± 12.1
	Columbidae (5)		52.1 ± 0.62	
	Dendrocolaptidae (8)		52.5 ± 0.72	
	Fringillidae (1)		51.85	
	Onychorhynchidae (1)		51.85	
	Passerellidae (1)		51.85	
	Pipridae (10)		51.8 ± 0.00	
	Thraupidae (5)		51.8 ± 0.00	
	Trochilidae (2)		51.8 ± 0.00	
	Turdidae (15)		52.3 ± 0.70	
	Typhlopidae (1)		45.4	
	Tyraniidae (1)		51.8	
Itambé	Cricetidae (18)	Atlantic Forest	57.4 ± 12.4	56.4 ± 11.8
	Didelphidae (36)		56.2 ± 11.6	
Itapetinga	Cricetidae (38)	Atlantic Forest	72.1 ± 11.2	68.9 ± 13.9
	Didelphidae (84)		67.4 ± 14.8	
Lajedo do Tabocal	Cricetidae (56)	Atlantic Forest	28.7 ± 7.89	30.6 ± 8.54
	Didelphidae (10)		36.4 ± 9.49	
	Echimidae (14)		34.2 ± 7.91	
Maracás	Cricetidae (46)	Atlantic Forest	58.6 ± 11.1	64.7 ± 21.4
	Cricetidae (77)	Caatinga	63.5 ± 24.8	
	Didelphidae (1)	Atlantic Forest	40.5	
	Didelphidae (34)	Caatinga	72.0 ± 21.6	
	Echimidae (16)		76.3 ± 18.0	
	Muridae (1)	Atlantic Forest	40.5	
Mascote	Cricetidae (8)	Atlantic Forest	85.1 ± 11.7	87.2 ± 9.36
	Didelphidae (11)		88.7 ± 7.51	
Mata de São João	Bradypodidae (11)	Atlantic Forest	8.52 ± 2.43	8.52 ± 2.42
Morro do Chapéu	Phyllodactylidae (1)	Atlantic Forest	41.1	41.1 ± 0.00
	Scincidae (1)		41.1	
	Tropiduridae (2)		41.1 ± 0.00	
Una	Callithrichidae (9)	Atlantic Forest	48.4 ± 0.00	12.0 ± 14.4
	Columbidae (1)		5.61	
	Cricetidae (6)		10.3 ± 10.6	
	Dendrocolaptidae (10)		5.61 ± 0.00	
	Didelphidae (9)		11.1 ± 6.84	
	Furnariidae (1)		5.61	
	Grallariidae (1)		5.61	
	Picidae (1)		5.61	
	Pipridae (23)		5.61 ± 0.00	
	Rhynchocyclidae (1)		5.61	
	Turdidae (9)		5.58 ± 0.09	
	Tyraniidae (1)		5.61	
Uruçuca	Dendrocolaptidae (6)	Atlantic Forest	15.4 ± 0.00	15.4 ± 0.00
	Pipridae (1)		15.4 ± 0.00	
	Turdidae (8)		15.4 ± 0.00	
	Tyraniidae (2)		15.4 ± 0.00	

**Table 3:** Frequencies of captured wildlife species at each sample site in Bahia State, Brazil.

Relative Frequency (%)	Absolute Frequency (*n*)	Species	Family	Class
14.1	95	*Didelphis albiventris*	*Didelphidae*	Mammalia
10.1	68	*Necromys lasiurus*	*Cricetidae*	Mammalia
9.20	62	*Calomys expulsus*	*Cricetidae*	Mammalia
6.53	44	*Cerradomys vivoi*	*Cricetidae*	Mammalia
5.79	39	*Marmosops incanus*	*Didelphidae*	Mammalia
4.75	32	*Hylaeamys seuanezi*	*Cricetidae*	Mammalia
4.60	31	*Gracilinanus agilis*	*Didelphidae*	Mammalia
3.41	23	*Rhipidomys mastacalis*	*Cricetidae*	Mammalia
3.41	23	*Wiedomys pyrrhorhinos*	*Cricetidae*	Mammalia
3.12	21	*Leontopithecus chrysomelas*	*Callithrichidae*	Mammalia
2.67	18	*Turdus leucomelas*	*Turdidae*	Avian
2.67	18	*Thrichomys aff laurentius*	*Echimidae*	Mammalia
2.37	16	*Marmosa murina*	*Didelphidae*	Mammalia
1.93	13	*Turdus rufiventris*	*Turdidae*	Avian
1.78	12	*Ceratopipra rubrocapilla*	*Pipridae*	Avian
1.63	11	*Bradypus torquatus*	*Bradypodidae*	Mammalia
1.63	11	*Coleodactylus meridionalis*	*Sphaerodactylidae*	Reptilia
1.34	9	*Dixiphia pipra*	*Pipridae*	Avian
1.19	8	*Xiphorhynchus fuscus*	*Dendrocolaptidae*	Avian
1.19	8	*Oligoryzomys nigripes*	*Cricetidae*	Mammalia
1.19	8	*Trinomys albispinus*	*Echimidae*	Mammalia
1.04	7	*Dendrocincla turdina*	*Dendrocolaptidae*	Avian
1.04	7	*Manacus manacus*	*Pipridae*	Avian
1.04	7	*Mus musculus*	*Cricetidae*	Mammalia
1.04	7	*Oligoryzomys stramineus*	*Cricetidae*	Mammalia
0.89	6	*Machaeropterus regulus*	*Pipridae*	Avian
0.89	6	*Cryptonanus agricolai*	*Didelphidae*	Mammalia
0.74	5	*Leptotila rufaxilla*	*Columbidae*	Avian
0.59	4	*Glyphorynchus spirurus*	*Dendrocolaptidae*	Avian
0.59	4	*Trinomys* sp.	*Echimidae*	Mammalia
0.45	3	*Monodelphis americana*	*Didelphidae*	Mammalia
7.01	48	Others	-	Avian/Mammalia/Reptilia
100	674	Total		

## Data Availability

No new data were created or analyzed in this study. Data sharing is not applicable in this study.

## Supplementary Materials

The following supporting information can be downloaded at: https://www.mdpi.com/article/10.3390/ani14010021/s1, Table S1: Absolute and relative frequency of captured and sampled wildlife, characterized by class, season, biome, locomotion, and diet; Table S2: Evaluated parameters of captured and sampled wildlife in the municipalities of Bahia State, Brazil; Table S3: Frequency of the feeding habits of the captured and sampled wildlife.

## References

[1] Rock M., Buntain B.J., Hatfield J.M., Hallgrímsson B. (2009). Animal–Human Connections, “One Health”, and the Syndemic Approach to Prevention. Soc. Sci. Med..

[2] De Oliveira Iovine R.O., Dejuste C., Miranda F., Filoni C., Bueno M.G., de Carvalho V.M. (2015). Isolation of *Escherichia coli* and *Salmonella* spp. from Free-Ranging Wild Animals. Braz. J. Microbiol..

[3] Tompkins D.M., Dunn A.M., Smith M.J., Telfer S. (2011). Wildlife Diseases: From Individuals to Ecosystems. J. Anim. Ecol..

[4] Siembieda J.L., Kock R.A., McCracken T.A., Newman S.H. (2011). The Role of Wildlife in Transboundary Animal Diseases. Anim. Health Res. Rev..

[5] Hilbert F., Smulders F.J.M., Chopra-Dewasthaly R., Paulsen P. (2012). *Salmonella* in the Wildlife-Human Interface. Food Res. Int..

[6] López-Islas J.J., Méndez-Olvera E.T., Martínez-Gómez D., López-Pérez A.M., Orozco L., Suzan G., Eslava C. (2022). Characterization of *Salmonella* spp. and *E. coli* Strains Isolated from Wild Carnivores in Janos Biosphere Reserve, Mexico. Animals.

[7] Rubini S., Ravaioli C., Previato S., D’Incau M., Tassinari M., Guidi E., Lupi S., Merialdi G., Bergamini M. (2016). Prevalence of *Salmonella* Strains in Wild Animals from a Highly Populated Area of North-Eastern Italy. Ann. Ist. Super. Sanità..

[8] Millan J., Aduriz G., Moreno B., Juste R.A., Barral M. (2004). Salmonella Isolates from Wild Birds and Mammals in the Basque Country (Spain). Rev. Sci. Tech. OIE.

[9] Carraro P.E., Barbosa F.D.O., Benevides V.P., Casas M.R.T., Berchieri Junior A., Bürger K.P. (2022). Prevalence and Antimicrobial Resistance of *Salmonella* spp. Isolated from Free-Ranging Wild Boars in the State of São Paulo, Brazil. Cienc. Rural..

[10] Merkevičienė L., Butrimaitė-Ambrozevičienė Č., Paškevičius G., Pikūnienė A., Virgailis M., Dailidavičienė J., Daukšienė A., Šiugždinienė R., Ruzauskas M. (2022). Serological Variety and Antimicrobial Resistance in *Salmonella* Isolated from Reptiles. Biology.

[11] Fluit A.C., Schmitz F.-J. (2004). Resistance Integrons and Super-Integrons. Clin. Microbiol. Infect..

[12] Perveen N., Muzaffar S.B., Al-Deeb M.A. (2021). Exploring Human-Animal Host Interactions and Emergence of COVID-19: Evolutionary and Ecological Dynamics. Saudi J. Biol. Sci..

[13] Uelze L., Bloch A., Borowiak M., Grobbel M., Deneke C., Fischer M., Malorny B., Pietsch M., Simon S., Szabó I. (2021). What WGS Reveals about *Salmonella enterica* subsp. *enterica* in Wildlife in Germany. Microorganisms.

[14] IBGE, Instituto Brasileiro de Geografia e Estatística (2019). Biomas e Sistema Costeiro-Marinho Do Brasil: Compatível Com a Escala 1:250,000.

[15] Dos Santos E.J.E., Azevedo R.P., Lopes A.T.S., Rocha J.M., Albuquerque G.R., Wenceslau A.A., Miranda F.R., Rodrigues D.d.P., Maciel B.M. (2020). *Salmonella* spp. in Wild Free-Living Birds from Atlantic Forest Fragments in Southern Bahia, Brazil. BioMed Res. Int..

[16] Aabo S., Rasmussen O.F., Roseen L., Sørensen P.D., Olsen J.E. (1993). *Salmonella* Identification by the Polymerase Chain Reaction. Mol. Cel. Probes.

[17] Maciel B.M., Argôlo Filho R.C., Nogueira S.S.C., Dias J.C.T., Rezende R.P. (2010). High Prevalence of *Salmonella* in Tegu Lizards (*Tupinambis Merianae*), and Susceptibility of the Serotypes to Antibiotics. Zoonoses Public Health.

[18] Bauer A.W., Kirby W.M., Sherris J.C., Turck M. (1966). Antibiotic Susceptibility Testing by a Standardized Single Disk Method. Am. J. Clin. Pathol..

[19] CLISI, Clinical and Laboratory Standards Institute (2014). Performance Standards for Antimicrobial Susceptibility Testing; Twenty-Fourth Informational Supplement.

[20] Dohoo I., Martin W., Stryhn H.E. (2010). Veterinary Epidemiologic Research.

[21] Skov M.N., Madsen J.J., Rahbek C., Lodal J., Jespersen J.B., Jørgensen J.C., Dietz H.H., Chriél M., Baggesen D.L. (2008). Transmission of *Salmonella* between Wildlife and Meat-Production Animals in Denmark. J. Appl. Microbiol..

[22] Vogler B.R., Zurfluh K., Mattmann P., Schmitt K., Albini S. (2021). Low Occurrence of *Salmonella* spp. in Wild Birds from a Swiss Rehabilitation Centre. Vet. Rec. Open.

[23] Botti V., Navillod F.V., Domenis L., Orusa R., Pepe E., Robetto S., Guidetti C. (2013). *Salmonella* spp. and Antibiotic-Resistant Strains in Wild Mammals and Birds in North-Western Italy from 2002 to 2010. Vet. Ital..

[24] Skarżyńska M., Zając M., Kamińska E., Bomba A., Żmudzki J., Jabłoński A., Wasyl D. (2020). *Salmonella* and Antimicrobial Resistance in Wild Rodents—True or False Threat?. Pathogens.

[25] Dos Santos E.J.E., Lopes A.T.S., Maciel B.M., Bhonchal Bhardwaj S. (2022). Salmonella in Wild Animals: A Public Health Concern. Enterobacteria.

[26] Ribas A., Saijuntha W., Agatsuma T., Prantlová V., Poonlaphdecha S. (2016). Rodents as a Source of *Salmonella* Contamination in Wet Markets in Thailand. Vector Borne Zoonotic Dis..

[27] Williams S.H., Che X., Paulick A., Guo C., Lee B., Muller D., Uhlemann A.-C., Lowy F.D., Corrigan R.M., Lipkin W.I. (2018). New York City House Mice (*Mus Musculus*) as Potential Reservoirs for Pathogenic Bacteria and Antimicrobial Resistance Determinants. mBio.

[28] Mares M. (2017). Current Topics in Salmonella and Salmonellosis.

[29] Backhans A., Jacobson M., Hansson I., Lebbad M., Lambertz S.T., Gammelgård E., Saager M., Akande O., Fellström C. (2013). Occurrence of Pathogens in Wild Rodents Caught on Swedish Pig and Chicken Farms. Epidemiol. Infect..

[30] De Barros B.D.C.V., Chagas E.N., Bezerra L.W., Ribeiro L.G., Duarte Júnior J.W.B., Pereira D., da Penha Junior E.T., Silva J.R., Bezerra D.A.M., Bandeira R.S. (2018). Rotavirus A in Wild and Domestic Animals from Areas with Environmental Degradation in the Brazilian Amazon. PLoS ONE.

[31] Carusi L.C.P., Farace M.I., Ribicich M.M., Villafañe I.E.G. (2009). Reproduction and Parasitology of *Didelphis albiventris* (Didelphimorphia) in an Agroecosystem Landscape in Central Argentina. Mammalia.

[32] Casagrande R.A., Lopes L.F.L., Reis E.M.d., Rodrigues D.d.P., Matushima E.R. (2011). Isolation of *Salmonella enterica* in opossum (*Didelphis aurita* and *Didelphis albiventris*) of the São Paulo State, Brazil. Cienc. Rural..

[33] Reinthaler F.F., Posch J., Feierl G., Wüst G., Haas D., Ruckenbauer G., Mascher F., Marth E. (2003). Antibiotic resistance of *E. coli* in sewage and sludge. Water Res..

[34] Robinson T.P., Bu D.P., Carrique-Mas J., Fèvre E.M., Gilbert M., Grace D., Hay S.I., Jiwakanon J., Kakkar M., Kariuki S. (2016). Antibiotic resistance is the quintessential One Health issue. Trans. R. Soc. Trop. Med. Hyg..

[35] Wang J., Ma Z.B., Zeng Z.L., Yang X.W., Huang Y., Liu J.H. (2017). The role of wildlife (wild birds) in the global transmission of antimicrobial resistance genes. Zool. Res..

[36] Jechalke S., Heuer H., Siemens J., Amelung W., Smalla K. (2014). Fate and effects of veterinary antibiotics in soil. Trends Microbiol..

[37] Radhouani H., Silva N., Poeta P., Torres C., Correia S., Igrejas G. (2014). Potential impact of antimicrobial resistance in wildlife, environment and human health. Front. Microbiol..

[38] Parmar T.K., Rawtani D., Agrawal Y.K. (2016). Bioindicators: The Natural Indicator of Environmental Pollution. Front. Life Sci..

